# Ataxia-Telangiectasia Mutated Loss of Heterozygosity in Melanoma

**DOI:** 10.3390/ijms232416027

**Published:** 2022-12-16

**Authors:** Lorenza Pastorino, Bruna Dalmasso, Eleonora Allavena, Irene Vanni, Filippo Ugolini, Gianna Baroni, Michela Croce, Antonio Guadagno, Francesco Cabiddu, Virginia Andreotti, William Bruno, Gabriele Zoppoli, Lorenzo Ferrando, Enrica Teresa Tanda, Francesco Spagnolo, Chiara Menin, Rosaria Gangemi, Daniela Massi, Paola Ghiorzo

**Affiliations:** 1 Department of Internal Medicine and Medical Specialties, University of Genoa, 16132 Genoa, Italy; 2 Genetica dei Tumori Rari, IRCCS Ospedale Policlinico San Martino, 16132 Genoa, Italy; 3 Section of Anatomic Pathology, Department of Health Sciences, University of Florence, 50134 Florence, Italy; 4 Bioterapie, IRCCS Ospedale Policlinico San Martino, 16132 Genoa, Italy; 5 Anatomia Patologica, IRCCS Ospedale Policlinico San Martino, 16132 Genoa, Italy; 6 Clinica di Medicina Interna a Indirizzo Oncologico, IRCCS Ospedale Policlinico San Martino, 16132 Genoa, Italy; 7 Oncologia Medica 2, IRCCS Ospedale Policlinico San Martino, 16132 Genoa, Italy; 8 Immunology and Diagnostic Molecular Oncology Unit, Veneto Institute of Oncology, IOV-IRCCS, 35128 Padua, Italy

**Keywords:** germline variant, loss of heterozygosity, melanoma, ATM, susceptibility

## Abstract

ATM germline pathogenic variants were recently found enriched in high-risk melanoma patients. However, ATM loss of heterozygosity (LOH) has never been investigated in melanoma and, therefore, a causal association with melanoma development has not been established yet. The purpose of this study was to functionally characterize 13 germline ATM variants found in high-risk melanoma patients—and classified by in silico tools as pathogenic, uncertain significance, or benign—using multiple assays evaluating ATM/pATM expression and/or LOH in melanoma tissues and cell lines. We assessed ATM status by Immunohistochemistry (IHC), Western Blot, Whole-Exome Sequencing/Copy Number Variation analysis, and RNA sequencing, supported by Sanger sequencing and microsatellite analyses. For most variants, IHC results matched those obtained with in silico classification and LOH analysis. Two pathogenic variants (p.Ser1135_Lys1192del and p.Ser1993ArgfsTer23) showed LOH and complete loss of ATM activation in melanoma. Two variants of unknown significance (p.Asn358Ile and p.Asn796His) showed reduced expression and LOH, suggestive of a deleterious effect. This study, showing a classic two-hit scenario in a well-known tumor suppressor gene, supports the inclusion of melanoma in the ATM-related cancer spectrum.

## 1. Introduction

The ataxia-telangiectasia mutated (ATM) gene encodes a serine/threonine kinase protein that plays a critical role in the activation of cellular responses to DNA double-strand breaks through downstream phosphorylation of central players in the DNA damage-response pathways, including BRCA1, p53, and Chk2 [1].

Germline inactivation of both copies of the ATM gene causes Ataxia-Telangiectasia (A–T), an autosomal recessive disorder characterized by progressive cerebellar degeneration, oculocutaneous telangiectasias, immunodeficiency, and susceptibility to cancer, mainly leukemias and lymphomas [2]. Studies of A-T families showed that heterozygous ATM pathogenic variant (PV) carriers are at increased risk of breast cancer [3]. Since then, other malignancies, such as pancreatic cancer, have been linked to ATM through studies conducted on cancer cohorts. Indeed, ATM PV carriers are primarily identified outside of an A-T familial context through multigene panel testing. Therefore, cancer risks associated with ATM variants are of relevance to genetic counselling and cancer risk management of a much larger population [4]. However, the hypervariability due to the large size of this gene, and the fact that several of the identified variants are variants of uncertain significance (VUSs), pose a challenge to the inclusion of additional tumors in the ATM-related cancer spectrum.

In a recent multicentric international study on 2105 melanoma cases, we associated germline heterozygous variants in ATM with melanoma risk, therefore proposing ATM, which was previously established as a melanoma genome-wide association study (GWAS) hit [5,6], as a melanoma intermediate-risk gene [7].

However, loss of heterozygosity of the ATM gene as described, for example, for breast cancer [8,9] has never been investigated in melanoma. Therefore, a causal association with melanoma development has not been established yet. No histopathological and molecular features have been studied in melanomas developed by carriers of germline ATM PV or VUS. Therefore, the ATM impact on melanoma predisposition is unclear.

Somatic ATM mutations rarely occur in melanoma (2.6%), and the literature on ATM protein expression and its clinical significance in melanoma is scant, although Bhandaru et Al. suggested that both loss and gain in phosphorylated ATM (pATM) expression were unfavorably associated with survival in melanoma patients [10].

Based on these premises, the purpose of this study was to functionally characterize ATM variants classified as PV or VUS by in silico tools found in the germline of melanoma patients by determining ATM and pATM expression and assessing loss of heterozygosity (LOH) in patients’ derived melanoma tissues and cell lines.

## 2. Results

### 2.1. Classification and Distribution of Germline ATM Variants

**Figure 1:**
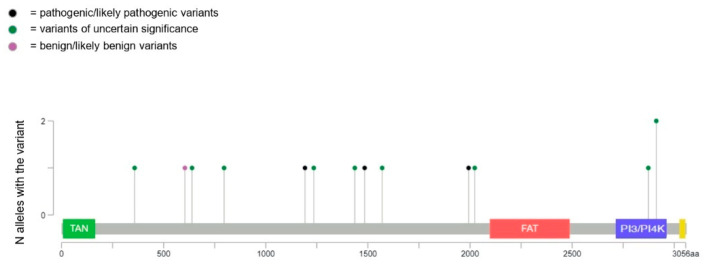
The lollipop plot shows the distribution of the ATM variants included in this study. TAN = Tel1/ATM N-terminal motif domain; FAT = FRAP–ATM–TRRAP domain; PI3/PI4K = phosphatidylinositol 3-kinase/phosphatidylinositol 4-kinase-related kinase domain.

Three germline pathogenic variants (PVs), eight variants of unknown significance (VUSs), and one benign (B) variant (Figure 1), classified according to the ACMG criteria [11], were studied by comparing them with an ATM wild-type control. The p.Met1484fs PV, which we previously described in patients diagnosed with multiple primary melanoma and pancreatic cancer [7,12], has never been detected in patients with AT, but was found in a patient with Metastatic Castration-resistant Prostate Cancer [13].

The p.Ser1135_Lys1192del and p.Ser1993ArgfsTer23 PV, as well as the p.Gly2023Arg likely benign (LB) variant, were previously described in either homozygous or compound heterozygous A-T patients [14,15], as well as in breast cancer patients [16,17,18]. Both the patient carrying the p.Ser1135_Lys1192del PV in this study and two other carriers not included in this study were found to share a common recurrent haplotype at the ATM locus already identified in Italian AT patients [14], suggesting a possible founder effect in this ATM variant. Eleven variants were found in high-risk melanoma patients reported in our previous studies [7,12], whereas one variant, the p.Asn796His, has never been described in melanoma but has been described in breast cancer [18,19]. Conversely, the p.Asp639Gly variant has only been reported once (https://www.ncbi.nlm.nih.gov/clinvar/RCV001013687/, accessed on 16 June 2022) and classified as VUS, but no associations with cancers or cancer-related syndromes have been observed, to our knowledge. The p.His 1436Tyr has been described in breast cancer [20] and prostate cancer [8], and the p.Val1570Ala has been described in breast and pancreatic cancer and in chronic lymphocytic leukemia [21,22,23]. For additional information on personal/family history of cancer and co-segregation analysis, see Table 1.

Figure one shows the distribution of germline ATM variants included in this study. Of all the analyzed variants, two VUS, p.Val2873Ile and p.Arg2912Gly, are located in the kinase domain. The latter was described in a breast cancer patient [18] and has been found to result in a partial impairment of ATM kinase activity in a previous publication [24]. The remaining variants are distributed outside the gene’s functional domains, with no particular clustering.

### 2.2. ATM Immunohistochemistry and LOH Assessment

IHC expression showed high concordance with the in silico classification of germline variants (Table 2).

**Figure 2:**
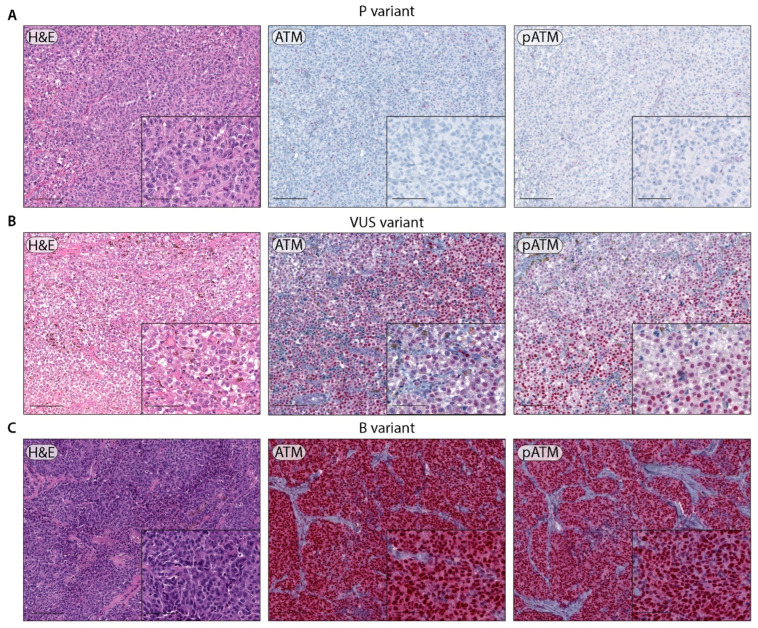
Representative images of ATM and pATM expression by immunohistochemistry. (**A**) Metastatic melanoma with pathogenic ATM variant (p.Ser1993ArgfsTer23), showing a lack of ATM and pATM nuclear expression in tumor cells (magnification 10X, inset 20X; scale bars 200 μm and 100 μm, respectively). (**B**) Metastatic melanoma with ATM variant of uncertain significance (p.Asn796His), showing partial conservation of nuclear expression for ATM and for pATM (magnification 10X, inset 20X; scale bars 200 μm and 100 μm, respectively). (**C**) Skin melanoma metastasis with benign ATM variant (p.Pro604Ser) showing nuclear ATM and pATM staining (magnification 10×, inset 20×; scale bars 200 μm and 100 μm, respectively).

Indeed, in 2 out of 3 patients carrying a germline PV (Mel_1 and Mel_3), loss of ATM and pATM expression was observed (Mel_3, Figure 2A) and correlated with the presence of the mutated allele at a high frequency as detected by DNA sequencing in the melanoma tissue, as well as in breast cancer tissue from one of the two patients (Table 2).

Mel_1, carrying the p.Ser1135_Lys1192del PV, showed loss of ATM and pATM expression in both breast cancer tissue and melanoma. According to previous studies conducted on non-melanoma cancers, this variant results in the skipping of ATM exons 26–27, with impaired/absent protein function [25,26]. LOH was confirmed by microsatellite analysis in both cases (Table 2). Therefore, expression and LOH analysis support the in silico classification of two out of three PVs as pathogenic for melanoma (p.Ser1993ArgfsTer23, p.Ser1135_Lys1192del).

**Figure 3:**
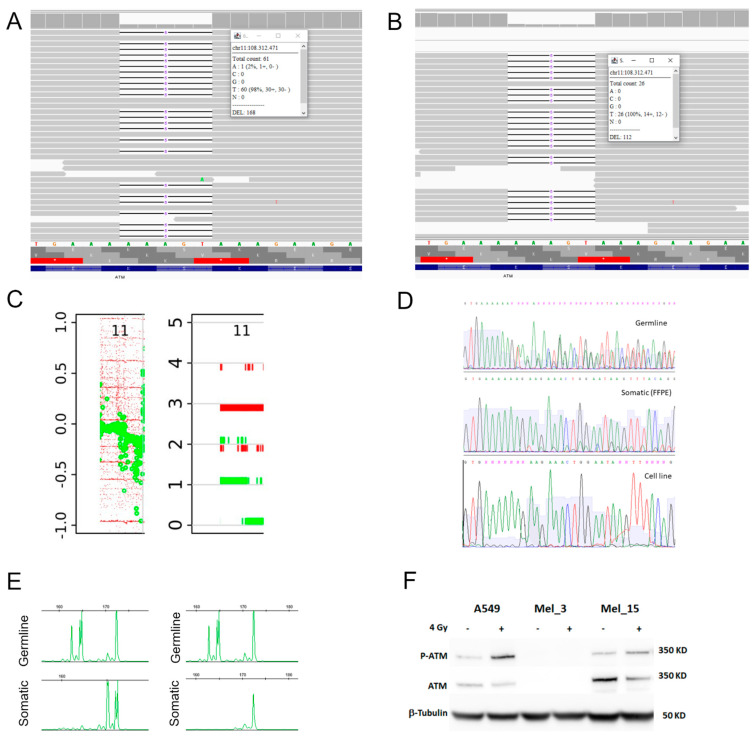
Multiple assays showing loss of heterozygosity of the ATM p.Ser1993ArgfsTer23 PV in Mel_3. (**A**) WES showing prevalence of the mutant allele (c.5979_5983del), with an AF of 74%. (**B**) RNA sequencing showing a mutant allele (c.5979_5983del) with a frequency of 94%. (**C**) The LogR plot (left) derived from WES analysis shows the presence of CNV: a region on chromosome 11q that encompasses the ATM gene (chr11: 100650080-115623321) shows copy-number loss; in the ploidy plot (right), the same region of the tetraploid sample contains three copies of a single allele (red bar), whereas the other allele is deleted (green bar). (**D**) Sanger sequencing showing the heterozygous germline allele (top), partial loss of the WT allele (middle) in the FFPE sample, and the complete loss of the WT allele in the patient’s derived melanoma cell line (bottom). (**E**) Microsatellite analysis showing LOH in one of the FFPE samples and in the patient’s derived melanoma cell line: D11S2179 LOH in tumor is determined by comparing the peak-height ratio of blood sample (top) with those of tumor (bottom left) and cell line (bottom right) DNA. (**F**) Western blotting. ATM protein expression was tested on A549, a NSCLC cell line expressing functional ATM, and on two primary cell lines derived from metastatic cutaneous melanoma biopsies, Mel_3 and Mel_15. A549 and Mel_15 cells express ATM and upmodulate the expression of its phosphorylated form upon irradiation at 4 Gray. Conversely, the Mel_3 cell line does not express either ATM or p-ATM. Cropped images are obtained from the same blot subsequently probed with the different antibodies. Molecular weight markers are shown. Abbreviations: AF = Allele Frequency. FFPE = formalin fixed paraffin embedded.

Moreover, in Mel_3, whole-exome sequencing (WES) was performed on DNA extracted from fresh-frozen melanoma tissue (cutaneous metastasis), and the p.Ser1993ArgfsTer23 variant was found at an allele frequency of 74%. Considering that the aberrant cell fraction was 80% in this sample, we could deduce that the pathogenetic variant is present in all tumor cells (Figure 3A). The same results were obtained by analyzing an additional fresh-frozen metastatic sample obtained from the same patient (muscle metastasis).

Conversely, Mel_2, bearing the p.Met1484fs PV, retained ATM and pATM expression by immunohistochemistry. However, no further tissues were available for LOH validation. Among the ten samples carrying VUSs, 8 retained ATM and pATM expression, and in one of them (Mel_12) in which microsatellite analysis was performed, the IHC results were consistent with the absence of LOH. Conversely, in two samples (Mel_4 and Mel_6), ATM and pATM expressions were equal to or lower than 50% (Figure 2B) and LOH was confirmed in one of them (Mel_6) by microsatellite analysis. Sanger sequencing, when performed, confirmed LOH analysis data (see Table 2). As expected, the samples carrying the p.Pro604Ser benign and the p.Gly2023Arg likely benign variants retained both ATM and pATM expression in immunohistochemistry, in line with the in silico classification (Figure 2C).

### 2.3. Western Blotting, DNA, and RNA Sequencing in Melanoma Tissue and Patient-Derived Melanoma Cell Lines

In the sample with the p.Ser1993ArgfsTer23 PV (Mel_3), LOH was confirmed by RNA sequencing conducted on two different metastatic melanoma samples, in which the mutant allele frequency was 94% (Figure 3B) and 82%. Moreover, CNV analysis of WES data revealed a whole-gene deletion on the WT ATM allele in both samples, which was confirmed by Sanger sequencing and microsatellite analysis, showing loss of the WT allele in both tissues and in the melanoma cell line (Figure 3C–E). Loss of ATM and pATM expression was also revealed by WB performed on the cell line established from the patient’s fresh tissue (Figure 3F).

In addition, we analyzed a melanoma tissue sample from one patient (Mel_15) with a somatic ATM variant classified as tier 1 [27] and no germline variants. We found the variant in a heterozygous status by both WES and Sanger sequencing in multiple lesions from the same patient, and used it as a heterozygous control. In this sample, IHC showed high expression of ATM and pATM (Figure 3F). WB confirmed this result as cells expressed ATM and upmodulated the expression of its phosphorylated form upon irradiation at 4 Gray (Figure 3F).

## 3. Discussion

The increasing availability of novel therapies that exploit vulnerabilities in the DNA Damage Response (DDR) machinery has prompted the investigation of DDR alterations in multiple cancers, both at the germline and the somatic level. Indeed, as for other cancers, loss of mechanisms maintaining genomic integrity could be one of the critical events in the pathogenesis of melanoma. Recently, germline PVs in the ATM gene have been found enriched in high-risk melanoma patients, and have been reported with a frequency of 1.06% in high-risk Italian melanoma patients [28]. However, the association of ATM with melanoma needs functional assessment starting from LOH analysis to definitively include this tumor in the ATM-related cancer spectrum.

With this study, we wanted to determine the role of ATM disruption in melanoma development by integrating multiple in vitro assays. In 2 out of 3 patients carrying PVs, the ATM protein was absent in melanoma tissue, hence supporting the hypothesis that loss of ATM tumor suppressor function is involved in melanoma development. For one of these variants—the founder p.Ser1135_Lys1192del—our results confirm, in melanoma, the negative effect on the ATM protein reported in previous studies [25,26]. In the third patient with a PV, however, ATM expression and kinase activity were apparently unimpaired according to IHC. Unfortunately, however, we could not confirm or contradict this result with a second technique. As expected, the second allele was retained in the analyzed tumor samples when the germline variant was classified as benign. VUS analysis allowed us to explore not only the effect of ATM on melanoma, but also the effect of the variant itself on ATM function. Indeed, a growing number of in silico tools are available to classify genetic variants. However, although those tools can predict pathogenicity through several sophisticated algorithms, in vitro/in vivo experiments are ultimately required to obtain conclusive proof of the nature of a variant. For the large majority of the VUSs analyzed in this study, we did not observe loss of the ATM protein in tumor tissue. Therefore, we cannot provide conclusive information on the pathogenicity of these variants. In the tumor samples of two patients with VUS, however, ATM expression was ≤50%, suggesting that these two germline variants represented the first of two hits that contributed to ATM deficiency and melanoma development. These data suggest that these two missense variants should probably be included in Class 4/5 of pathogenicity. However, further research is needed in order to reclassify them. In fact, our study had some limitations. For instance, although we employed multiple techniques to study the abundance of ATM in all tumor samples, the paucity of available material prevented us from applying all the techniques to each sample. However, for the p.Ser1993ArgfsTer23 PV, we were able to integrate concordant results obtained from seven different assays (Figure 3) in multiple tumor tissues and in an established melanoma cell line from the same patient.

Taken together, our results provide, for the first time, experimental data in support of the role of ATM impairment in melanoma development, showing a classic two-hit scenario in a well-known tumor suppressor gene. This is of particular relevance, as the identification of ATM as a melanoma-causative gene has potentially multiple clinical implications. Indeed, the inclusion of ATM in melanoma multi-gene diagnostic panels would increase the number of high-risk individuals identified through genetic testing who could benefit from dermatologic surveillance.

Moreover, this work provides the rationale for further studies, some of which are already ongoing, with the aim of exploring the potential actionability of DDR genes in selected melanoma patients. Specifically, in germline carriers, the deficiency of ATM and its related pathways could be exploited for personalized anticancer treatment approaches.

## 4. Materials and Methods

### 4.1. Selection of ATM Variant Carriers and Their Tumors

Eighteen tissue samples were retrieved for the present study from fourteen individuals belonging to the Genoa (N = 16) and Padua (N = 2) cohort of high-risk individuals carrying germline heterozygous ATM variants for whom melanoma tissue (and/or breast cancer and dysplastic naevus in two cases) was available at the Institutional pathology departments. In addition, one junctional nevus from a healthy individual carrying a heterozygous germline ATM variant (CTRL_1) and one melanoma tissue from a sporadic melanoma patient, a carrier of a heterozygous somatic ATM variant from which we established a melanoma cell line, were also included as controls (Mel_15). Heterozygous germline variants were classified according to the ACMG guidelines [11] and a subset had been previously described in the germline of melanoma patients [7,12] (Table 1).

### 4.2. Establishment of Melanoma Cell Lines

Human melanoma samples were obtained from patients selected at the IRCCS Ospedale Policlinico San Martino under local Institutional-Review-Board-approved protocols (CER Liguria: 046REG2017). Patient biopsies were placed in a sterile conical tube containing NaCl 0.9% and 1% antibiotic and antimycotic on wet ice and handled no later than 4 h after resection. Upon arrival, biopsies were manually minced using a sterile scalpel and dissociated using 70 µm cell strainers (Miltenyi Biotec) at room temperature. Cells were cultured in RPMI 10%FBS, 1%HEPES, 1%Na Pyruvate, 2%Glutamine, 1%Pen/Strep, and 1% antimycotic and stabilized as culture after the 8th passage. Positivity for melanoma antigens was assessed by flow cytometry (FACSCalibur; Becton Dickinson, East Rutherford, NJ, USA) in Mel_3 and Mel_15 cells using anti-CD146 (BD Pharmingen, cod 550315) and anti-MCSP (Miltenyi Biotec, cod 130-091-225) monoclonal antibodies.

A549, a NSCLC cell line expressing functional ATM, was purchased from the ICLC cell bank of IRCCS Ospedale Policlinico San Martino and cultured in RPMI 10%FBS, 2%Glutamine, and 1%Pen/Strep.

### 4.3. Irradiation Conditions

Cells (25 × 10^4^/mL in 4 mL) were irradiated (4 Gy) using IBL 437C (CIS Bio International). Cells were centrifuged and resuspended in fresh medium and kept at 37 °C for 1 h, then centrifuged and cell pellets lysed.

### 4.4. Immunohistochemistry of Clinical Specimens and Analysis of ATM and P-ATM Staining

Formalin-fixed paraffin-embedded (FFPE) 3 µm tissue sections were prepared for immunohistochemical analysis. Sample processing was performed with an automated immunostainer (Ventana Discovery ULTRA, Ventana Medical Systems, Tucson, AZ, USA). Sections were deparaffinized in EZ prep (#950–102; Ventana Medical Systems, Tucson, AZ, USA), and antigen retrieval was achieved by incubation with cell-conditioning solution 1 (CC1) for 64 min (#950–124; Ventana Medical Systems, Tucson, AZ, USA). Sections were then incubated with the following primary antibodies: anti-ATM (#ab32420 recombinant, clone Y170, 1:100, rabbit monoclonal, Abcam, Cambridge, UK); anti-P-ATM (#ab81292 recombinant, clone EP1890Y, 1:100, rabbit monoclonal, Abcam, Cambridge, UK). The signal was developed with anti-rabbit UltraMap RED detection kit (Ventana Medical Systems, Tucson, AZ, USA). Sections were counterstained with hematoxylin (Ventana Medical Systems, Tucson, AZ, USA). Stained tissue sections were digitally scanned at ×400 magnification with Aperio AT2 (Leica Biosystems, Wetzlar, DE, USA) into whole-slide digital images (WSI). Each SVS format file was imported into the HALO Link^®^ (Indica Labs, Albuquerque, NM, USA) image management system.

ATM and P-ATM immunostaining were semi-quantitatively evaluated based on the percentage of immunoreactive positive tumor cells with nuclear labeling per total number of tumor cells. For each slide, a score from 0 to 100% of positivity was assigned.

### 4.5. DNA Extraction

DNA samples were extracted from tumor-enriched areas using either the Genomic DNA FFPE One-Step Kit for Diatech MagCore^®^ HF16Plus extractor (RBC Bioscience, New Taipei City, Taiwan) or the QIAamp DNA FFPE Tissue kit (Qiagen, Valencia, CA, USA) and/or the DNeasy Blood & Tissue Kit for DNA from fresh tissue (FT) or primary melanoma cell lines, according to the manufacturer’s instructions.

### 4.6. Sanger Sequencing

Sanger Sequencing (SS) was used to confirm the presence of germline variants in the selected tissues/cell lines as previously described [29,30,31]. The sequencing products were separated by capillary electrophoresis (ABI 3130XL Genetic Analyzer, Applied Biosystems, Waltham, MA, USA) according to the manufacturer’s instructions.

### 4.7. Whole-Exome Sequencing (WES) and Copy Number Variation (CNV) Analysis

Genomic DNA from peripheral blood and somatic DNA from fresh tissue were subjected to whole-exome sequencing (WES) at a coverage of 100X and 300X, respectively. Nextera Flex for Enrichment solution (Illumina, San Diego, CA, USA) combined with ‘SureSelect Human All Exon V7’ probes (Agilent, Santa Clara, CA, USA) was used for library preparation and exome enrichment, targeting 50 Mb of human exonic content. All samples were quantified and quality-tested using the Qubit 2.0 Fluorometer (Invitrogen, Carlsbad, CA, USA) and Agilent 2100 Bioanalyzer (Agilent Technologies, Santa Clara, CA, USA). Libraries were sequenced on a NovaSeq 6000 (Illumina, San Diego, CA, USA) in 150 paired-end mode. Raw data were first processed for both format conversion and de-multiplexing by the Bcl2Fastq 2.0.2 version of the Illumina pipeline (https://support.illumina.com/content/dam/illumina-support/documents/documentation/software_documentation/bcl2fastq/bcl2fastq2-v2-20-software-guide-15051736-03.pdf, accessed on 1 October 2021). Adapter sequences were masked with Cutadapt v1.11 from raw fastq data using the following parameters: --anywhere (on both adapter sequences) --overlap 5 --times 2 --minimum-length 35 --mask-adapter [32]. Subsequently, Illumina DRAGEN Germline 3.5.7 and Somatic Pipelines 3.5.7 were used to map reads to the GRCh38/hg38 assembly and identify germline and somatic tumor/normal matched pair variants, respectively (https://emea.illumina.com/products/by-type/informatics-products/basespace-sequence-hub/apps/dragen-germline.html; https://emea.illumina.com/products/by-type/informatics-products/basespace-sequence-hub/apps/edico-genome-inc-dragen-somatic-pipeline.html, accessed on 1 October 2021). Variants were functionally annotated by Annovar [33]. CNVkit 0.9.7 was used to detect somatic Copy Number Alterations (CNAs) [34]. Tumor purity, ploidy, and copy number profiles were assessed using the ASCAT pipeline within the R computational environment [35,36].

### 4.8. RNA Sequencing

RNA from fresh tissue was extracted by the Tissue Lyser plus Maxwell^®^ RSC simplyRNA Tissue Kit (AS1340 Promega, Southampton, UK) in accordance with the manufacturer. Total RNA concentration was quantified by the Qubit^®^ 2.0 Fluorometer (Invitrogen) using Qubit™ RNA High-Sensitivity (HS) Assay Kits (ThermoFisher, Waltham, MA, USA). The RNA library was prepared using the Illumina Stranded Total RNA Prep with Ribo-Zero Plus (Illumina Inc., San Diego, CA, USA). Briefly, after ribosomal and globin RNA depletion, RNA was fragmented and denatured, and cDNA synthesized. Then, the 3′ ends were adenylated, and anchors ligated, followed by a PCR amplification step to add the index-adapter sequences. After clean-up and final amplification of the dual indexed libraries, the quality of the libraries was assessed by the 2100 Bioanalyzer with a High-Sensitivity DNA chip (Agilent), and libraries concentration was assessed with a High-Sensitivity Assay on a Qubit 4 fluorometer (ThermoFisher). Libraries were then pooled at 1.4 nM and paired-end-sequenced (2 × 150) on a NovaSeq 6000 Sequencing System instrument (Illumina Inc., San Diego, CA, USA) with the addition of 1% PhiX. Run metrics were 85.13% PF (clusters Passing Filter) and 91.02 Q30. Sequencing reads were aligned to the reference genome (GENCODE GRCh38 version 33) using STAR v.2.7.3a in two-pass basic mode preventing multimappings.

### 4.9. LOH Status by Microsatellite Analysis

Tumor tissue (FT and/or FFPE and/or melanoma cell line) and matched blood DNA were evaluated in a subset of patients for which good-quality and -quantity somatic DNA was available by using a PCR-based LOH assay with five fluorescence-labeled microsatellite markers (namely D11S1816, D11S1819, D11S2179, D11S1778, and D11S1294). Capillary electrophoresis was performed on the ABI 3100xL DNA analyzer. Raw electrophoretic data were analyzed with GeneMapper software to assess allele ratios. We considered LOH at the ATM locus when the allele ratio fell below 50% in the tumor DNA sample compared to the germline.

### 4.10. Western Blotting

Total proteins were extracted using Complete Lysis buffer containing proteases inhibitors (04719956001 Roche Diagnostics, Mannheim, Germany) and phosphatase inhibitors (04906845001 Roche). Protein lysates were separated by NuPage 4–12% Bis-Tris Gel (NP0335BOX Invitrogen) under reducing conditions. Western blotting (WB) was carried out according to standard techniques, with rabbit anti-pATM (phospho S1981) ab81292, anti-ATM ab32420, and anti-b-tubulin (HRP) ab21058 (Abcam, Cambridge, MA, USA). Immunoreactive proteins were detected by ECL Prime (GE Healthcare, RPN2232) and a chemiluminescence gel documentation and analysis system (MINI HD, UVITEC, Cambridge, UK).

## Figures and Tables

**Table 1:** Variants classification and clinical features.

Patient ID	Nucleotide Change	Protein Change	ACMG Classification	GnomAd NFE	Personal History of Cancer (Age of Diagnosis)	Other Cancer in Family (Age of Diagnosis)	Co-Segregation
Mel_1	c.3576G>A	p.Ser1135_Lys1192del	P (PM2,PP5)	3.52 × 10^−5^	CM (40) breast (46)	CM ^I^ (80)	YES
Mel_2	c.4451delT	p.Met1484fs	P (PVS1,PM2,PP5)	ND	3 CM (45), pancreas (50)	CM ^III^ (33), pancreas ^I^ (79)	YES
Mel_3	c.5979_5983del	p.Ser1993ArgfsTer23	P (PVS1,PM2,PP5)	1.76 × 10^−5^	CM (47)		
Mel_4	c.1073A>T	p.Asn358Ile	VUS (PM2)	ND	CM (64), endometrial (61)	CM ^I^ (81)	ND
Mel_5	c.1916A>G	p.Asp639Gly	VUS (PM2)	ND	4 CM (46)		
Mel_6	c.2386A>C	p.Asn796His	VUS (PM2)	8.80 × 10^−6^	CM (67)		
Mel_7	c.3704C>T	p.Pro1235Leu	VUS (PM2,PP3)	ND	CM (59), CRCC (56)	CM ^I^ (44), glioblastoma ^I^ (67)	ND
Mel_8	c.4306C>T	p.His1436Tyr	VUS (PM2)	4.41 × 10^−5^	2 CM (21)		
Mel_9	c.4709T>C	p.Val1570Ala	VUS (PM2)	7.30 × 10^−4^	6 CM (47)	CM ^II^ (na)	ND
Mel_10	c.8617G>A	p.Val2873Ile	VUS (PM2)	1.77 × 10^−5^	2 CM (40), prostate (52)	CM ^I^ (na), CM ^II^ (76)	ND
Mel_11	c.8734A>G	p.Arg2912Gly	VUS (PM2,PP3)	3.87 × 10^−4^	2 CM (29)		
Mel_12	c.8734A>G	p.Arg2912Gly	VUS (PM2,PP3)	3.87 × 10^−4^	6 CM (42), breast (51), lung (69)	CM ^I^ (36)	YES
Mel_13	c.1810C>T	p.Pro604Ser	B (BS2,BP6)	1.55 × 10^−3^	2 CM (42)	CM ^I^ (69), breast ^I^ (65), CM ^II^ (52)	ND
Mel_14	WT				CM (71)	CM ^III^ (43)	
CTRL_1	c.6067G>A	p.Gly2023Arg	LB (BP6)	2.30 × 10^−3^			

CM = cutaneous melanoma; P = pathogenic; VUS = variant of unknown significance; LB = likely benign; B = benign; ND = not determined; NA = not available; ^I^ = first degree; ^II^ = second degree; ^III^ = third degree.

**Table 2:** Histology, stage, IHC, and LOH assessment.

Patient ID	Histology	Stage Group	TNM Staging	Protein Change	ACMG Classification	ATM Expression by IHC (Ab32420)	p-ATM Expression by IHC (Ab81292)	LOH by Microsatellite Analysis/SS
Mel_1	Primary melanoma	IA	pT1a N0M0	p.Ser1135_Lys1192del	P	negative	negative	
	Breast Ductal Carcinoma	0	pTis N0 MX G2			negative	negative	YES
Mel_2	Primary melanoma	0	pTisN0M0	p.Met1484fs	P	99%	70%	
Mel_3	Melanoma—cutaneous metastasis	IV	-	p.Ser1993ArgfsTer23	P	1%	1%	YES
Mel_4	Primary melanoma	0	pTisN0M0	p.Asn358Ile	VUS	20%	15%	
Mel_5	Primary melanoma	0	pTisN0M0	p.Asp639Gly	VUS	85%	80%	
Mel_6	Melanoma—subperitoneal metastasis	IV	-	p.Asn796His	VUS	50%	50%	YES
Mel_7	Primary melanoma	IA	pT1a N0M0	p.Pro1235Leu	VUS	100%		
Mel_8	Dysplastic nevus		-	p.His1436Tyr	VUS	80%	75%	
Mel_9	Primary melanoma	IA	pT1a N0M0	p.Val1570Ala	VUS	99%		
Mel_10	Primary melanoma	IA	pT1a N0M0	p.Val2873Ile	VUS	100%	70%	
Mel_11	Dysplastic nevus			p.Arg2912Gly	VUS	95%	95%	
	Primary melanoma	0	pTisN0M0		VUS	99%	90%	
Mel_12	Primary melanoma	IA	pTxN0M0)	p.Arg2912Gly	VUS	95%	75%	NO
Mel_13	Primary melanoma	IA	pT1a N0M0	p.Pro604Ser	B	99%	95%	
	Primary melanoma	IA	pT1a N0M0			95%	90%	
Mel_14	Primary melanoma	IB	pT2aN0M0	-	-	95%	60%	NO
	Melanoma cutaneous metastasis	IV	-			100%	100%	
CTRL_1	Junctional nevus		-	p.Gly2023Arg	LB	99%	80%	

SS = Sanger sequencing; TNM = tumor, node, and metastasis staging system; P = pathogenic; VUS = variant of unknown significance; LB = likely benign; B = benign.

## Data Availability

Data available upon request.
